# Knowledge, curiosity, and aesthetic chills

**DOI:** 10.3389/fpsyg.2015.01546

**Published:** 2015-10-20

**Authors:** Félix Schoeller

**Affiliations:** Centre de Recherches sur les Arts et le Langage, École des Hautes Études en Sciences SocialesParis, France

**Keywords:** aesthetic chills, musicology, curiosity, emotion, knowledge

## Introduction

Chills are a muscular phenomenon best described as the sensation of coldness created by a rhythmic oscillating tremor of skeletal muscles. In humans, chills are sometimes associated with a positive hedonic process. In the present article, we shall refer to such an event as *aesthetic chills*. Aesthetic chills appear to be a universal emotional experience (see e.g., McCrae, 2007). In laboratory, this phenomenon has mainly been studied in the field of musicology using tonal music as an elicitor (e.g., Harrison and Loui, 2014) but chills may also be elicited by visual arts, literature, scientific research and religious practices (Schoeller, 2015). A large body of evidence seems to point in the direction that chills, shivers, and goose bumps reveal something fundamental about human beings and their relation to the natural world (for insightful review, see Maruskin et al., 2012). However, to this day, there exist no systematic account for these psychobiological events and available data are often contradictory—compare Halpern et al. (1986) to Grewe et al. (2007) or Goldstein (1980) to Panksepp (1995). In the present article, I propose a theory that reconciles these contradictions, review empirical evidence for such a theory and highlight problems to be addressed in future research.

## Aesthetic chills and knowledge-acquisition

The working hypothesis is that aesthetic chills correspond to a satisfaction of humans' internal drive to acquire knowledge about the external world and perceive objects and situations as meaningful. In humans, this need to explore and understand environmental conditions is a biological prerequisite for survival. In fact, even in lower animals, there is strong evidence for an antagonistic relationship between exploratory and eating responses (e.g., Chance and Mead, 1955). Theories in ethology and animal psychology suggest that exploratory behavior is motivated in higher mammals by an internal drive toward exploration (e.g., Harlow et al., 1950; White, 1959; Berlyne, 1960). In humans, knowledge-acquisition may usefully be modeled as an increase in a measure of similarity between mental representations and the external world (e.g., Grossberg, 1988; Perlovsky, 2006). Chills might correspond to an event where the total similarity of the cognitive system reaches its maxima (negative or positive), when the derivative of the function tends toward zero. In other words, chills would be elicited by an activity when (i) the subject fully understands external events and their future consequences or (ii) has no knowledge about them. That is, when nothing else than the current environment could have been predicted or when anything could happen and nothing is predictable by the subject. This would explain why chills are elicited by both extremely positive cues (aesthetic chills) and extremely negative cues (horror chills). In both cases, the correspondence between the subject's internal models and the external world reaches a peak value.

Chills might also be elicited in social situations and the theory should predict for such events as well. They are often reported in reaction to crowds (e.g., as in a public concert, movie theater, etc.), events where a group focuses on the same situation and shares a similar reaction (e.g., anger in a public manifestation, sadness at a funeral, etc.). In short, when our mental models and that of our fellowmen resonate (i.e., instances where our cognitive categories no longer appear to us as idiosyncratic). In all these cases, the subject finds evidence in the external world that her mental models are shared, functional and justified.

This theory fits the physiological data available. Music recruits neural systems similar to those known to respond specifically *to biologically relevant stimuli* (e.g., food or sex). In terms of functional anatomy, it has been shown that aesthetic chills correlate with decreases in regional cerebral blood flow in the amygdala and hippocampus (Blood and Zatorre, 2001). These neural structures play a crucial role in both reward and emotions (Berridge and Robinson, 1998). Chills may also be inhibited by the opioid antagonist naloxone (Goldstein, 1980) and animal studies in pharmacology provide extensive evidence that learning, curiosity, and retention are influenced by opioid peptides as well as by opiate drugs such as morphine and naloxone (see e.g., Martinez, 1981).

## Empirical research

There are at least three ways to study aesthetic chills experimentally: (i) one can investigate the *biological structure* at play, the neurophysiology of the phenomenon, its hormonal basis, etc., (ii) one can investigate the *psychological function* of chills, the relation between pleasure, curiosity and learning, etc., and finally (iii) one can investigate the *elicitor*, identify what kind of events causes negative/positive chills in most humans, the properties of such elicitors and their commonalities or differences. More empirical data is needed at all these levels and for all types of chills—musical chills, narrative chills, ceremonial chills, scientific chills, religious chills, etc.

The hypothesis that aesthetic emotions correspond to a satisfaction (or dissatisfaction) of humans' need for knowledge was tentatively confirmed in the context of musical emotions (Cabanac et al., 2013; Masataka and Perlovski, 2013). Testing this theory in the laboratory demands great precaution and for evident reasons it can prove quite difficult. One way to operationalize knowledge-acquisition is indeed to study *natural curiosity* (Berlyne, 1960). Experimentally, most of the difficulty in studying curiosity comes from the fact that, in higher mammals, most of the knowledge resulting from exploratory behavior is *latent* (e.g., Litman and Silvia, 2006) and thus rarely predictable. Furthermore, the construct itself is not yet fully understood and precise psychometric tools are not yet available—most of them focus on culture specific activities and do not address the problem of the adaptive structuration of knowledge (inherent to its acquisition).

The working hypothesis has been tested in four explorative studies conducted in a laboratory at the University of Copenhagen. In a battery of experiments, various aspects of the theory were investigated: (i) the relation between curiosity and chills (ii) the relation between meaning-making and the aesthetic experience, (iii) the narratology of chill-eliciting scenes, and (iv) the phenomenology of the aesthetic experience. Results seem to indicate that chills are positively correlated with curiosity and inhibited by incoherence, that a meaningful prime makes the aesthetic experience more pleasurable and that chills help subjects overcome cognitive dissonance of great strength. The stimulation used for the experiment was a film accompanied by Mozart's 23rd piano concerto, K488. The psychoacoustic properties of this stimulus are currently being analyzed and compared to that of other well-known chill-eliciting stimuli. At the narratological level, the properties of chill-eliciting scenes were analyzed and discussed in the light of redundancies found in the phenomenological descriptions provided by the participants (Schoeller, 2015). It was hypothesized from these results that chill-eliciting scenes facilitate the acceptance of fundamental cognitive conflicts. That is, contradictions involving pairs of cognitions of equal total resistance to change where the less resistant element may not be altered nor the amount of dissonance be reduced. I proposed that chill-eliciting scenes might provide subjects with *new cognitive elements* maintaining the total dissonance of the cognitive system at a rather low level.

## Conclusion

The study of chills offers the possibility for specialists in a wide range of different fields to work together and cognitive musicologists are at the forefront of this research. This will only be rendered possible by a shared language and there is a great need for a term that does not relate aesthetic chills to changes in temperature level (that is, a *universal term* that clearly distinguishes aesthetic chills from thermoregulatory chills). The empirical study of aesthetics in general and that of aesthetic chills in particular can shed light on some fundamental aspects of human nature and on the primordial role of music for the cognitive system. Furthermore, it might also lead to a better understanding of the psychobiology of humans' vital need for cognition.

### Conflict of interest statement

The author declares that the research was conducted in the absence of any commercial or financial relationships that could be construed as a potential conflict of interest.
